# Fatty Acid Profiles of Various Vegetable Oils and the Association between the Use of Palm Oil vs. Peanut Oil and Risk Factors for Non-Communicable Diseases in Yangon Region, Myanmar

**DOI:** 10.3390/nu10091193

**Published:** 2018-09-01

**Authors:** Wai Phyo Aung, Espen Bjertness, Aung Soe Htet, Hein Stigum, Virasakdi Chongsuvivatwong, Pa Pa Soe, Marte Karoline Råberg Kjøllesdal

**Affiliations:** 1 Department of Community Medicine and Global Health, Institute of Health and Society, Faculty of Medicine, University of Oslo, 0318 Oslo, Norway; espen.bjertness@medisin.uio.no (E.B.); aungsh@gmail.com (A.S.H.); hein.stigum@medisin.uio.no (H.S.); m.k.kjollesdal@medisin.uio.no (M.K.R.K.); 2 Procurement and Supply Division, Department of Public Health, Ministry of Health and Sports, Nay Pyi Taw 15011, Myanmar; 3 International Relations Division, Ministry of Health and Sports, Nay Pyi Taw 15011, Myanmar; 4 Epidemiology Unit, Prince of Songkla University, Hat Yai 90110, Thailand; cvirasak@gmail.com; 5 Department of Preventive and Social Medicine, University of Medicine 1, Yangon 11131, Myanmar; papasoe.jpn@gmail.com

**Keywords:** vegetable oils, palm oil, peanut oil, non-communicable diseases, Myanmar

## Abstract

The majority of vegetable oils used in food preparation in Myanmar are imported and sold non-branded. Little is known about their fatty acid (FA) content. We aimed to investigate the FA composition of commonly used vegetable oils in the Yangon region, and the association between the use of palm oil vs. peanut oil and risk factors for non-communicable disease (NCD). A multistage cluster survey was conducted in 2016, and 128 oil samples from 114 households were collected. Data on NCD risk factors were obtained from a household-based survey in the same region, between 2013 and 2014. The oils most commonly sampled were non-branded peanut oil (43%) and non-branded palm oil (19%). Non-branded palm oil had a significantly higher content of saturated fatty acids (36.1 g/100 g) and a lower content of polyunsaturated fatty acids (9.3 g/100 g) than branded palm oil. No significant differences were observed regarding peanut oil. Among men, palm oil users had significantly lower mean fasting plasma glucose levels and mean BMI than peanut oil users. Among women, palm oil users had significantly higher mean diastolic blood pressure, and higher mean levels of total cholesterol and triglycerides, than peanut oil users. Regulation of the marketing of non-branded oils should be encouraged.

## 1. Introduction

A large proportion of the vegetable oil on the global market is non-branded. The fatty acid content of non-branded oils is often not known, and the impact of non-branded oils on health remains to be elucidated. The fatty acid profile of vegetable oils influences the risk of non-communicable diseases (NCDs), including diabetes [1] and cardiovascular disease (CVD) [2]. A high intake of saturated fatty acids (SFA) has been associated with an increased risk of cardiovascular events [2]. Thus, reducing the dietary content of SFA is emphasized in CVD prevention [3]. The risk of diabetes is inversely associated with a higher intake of polyunsaturated fatty acid (PUFA), but no associations have been found with monounsaturated fatty acid (MUFA) or SFA [4]. Furthermore, a high intake of trans fat is associated with a higher risk of both diabetes and CVD [4,5]. The type of oil consumed is also related to NCD risk factors. A high intake of palm oil, compared to the intake of vegetable oils low in SFA, has been related to high levels of low-density lipoprotein (LDL) [6]. Vegetable oils rich in SFA, especially palmitic, myristic and lauric acid, increase blood cholesterol levels [7]. In terms of fatty acid composition, peanut oil is naturally free from cholesterol and trans fat [8]. Additionally, sesame oil plays a supportive role in hypertensive treatment [9], and vegetable oils rich in PUFA not only decrease diastolic blood pressure [10], but also lower fasting plasma glucose levels [11]. Moreover, total body fat, liver fat, and visceral fat accumulation increase with a higher intake of palmitic acid compared with PUFA [12].

NCDs are now the leading cause of death in most places in the world, with CVD being the leading cause, claiming 17.9 million lives each year [13]. The number of adults with diabetes increased from 108 million to 422 million between 1980 and 2014 [14]. In 2010, hypertension was one of the top three leading risk factors for the global burden of disease [15], and the number of people living with hypertension increased from 594 million to 1.13 billion between 1975 and 2015 [16].

In the Yangon region of Myanmar, the age-standardized prevalence of diabetes was 12.1% in urban areas and 7.1% in rural areas in 2014 [17]. The corresponding prevalences of hypertension were 34.5% in urban areas and 34.2% in rural areas [18], while those of hypercholesterolemia were 50.7% in urban areas and 41.6% in rural areas [19]. Vegetable oils are essential in the Myanmar diet [20] (‘Rice, oil, salt and medicine are a man’s basic needs’ is a saying in Myanmar). The annual consumption of edible oil per person in Myanmar is approximately 9.3 kg (10 kg in urban areas and 8.6 kg in rural areas) [20]. However, information on consumption patterns of vegetable oils, and the fatty acid content of the oils, is scarce in Myanmar. Peanut oil and sesame oil are the two major oils produced in Myanmar [20], while palm oil is the most commonly used oil in the country. However, in Lower Myanmar, including the Yangon region, peanut oil is the most frequently consumed oil [21]. Most of the palm oil is imported at a low price from Malaysia [20,21]. In Myanmar, palm oil is available in the market as pure palm oil, or else is mixed with peanut oil or sesame oil, and can be branded or non-branded [20].

The results from previous and future studies on the association between the types of oils used and health-related outcomes could be better interpreted if the FA contents of different types of oil were known. Therefore, the aim of this study was to identify the FA composition of vegetable oils consumed in households in the Yangon region of Myanmar, and to investigate the association between the two most commonly used types of cooking oil (palm oil and peanut oil) and NCD risk factors in study participants between the ages of 25 and 74.

## 2. Materials and Methods

To investigate the association between the type of oil used and NCD risk factors, we used data from a STEPS study of 25–74-year-old urban and rural inhabitants of the Yangon region, collected in 2013 and 2014, respectively. This study will hereafter be named the ‘STEPS 2014-study’. From this study, we had no information about the FA composition of the types of cooking oil used in households. Therefore, we additionally conducted a study in 2016, in which oil samples were collected from households in the same area as the STEPS 2014-study. This study will hereafter be named the ‘Oil 2016-study’. Buddhist monks and nuns, institutionalized people, military personal, and individuals who were too physically or mentally ill to participate were excluded from both studies.

### 2.1. Sampling Methodology

#### 2.1.1. STEPS 2014-Study

To assess the association between the intake of different vegetable oils and risk factors for NCD (body mass index (BMI), waist hip ratio (WHR), blood pressure (BP), fasting plasma glucose (FPG), total cholesterol (TC) and triglycerides (TG)), we used data from a previous household-based cross-sectional study conducted in the Yangon region between 2013 and 2014, which included 1372 participants between the ages of 25 and 74. The study followed the WHO STEP survey protocol [22], and its details have been described elsewhere [19,23].

The data on NCD risk factors was collected as follows: A measuring tape was used to measure the participants’ heights without footwear or headwear (measured to the nearest to 0.1 cm). The weight of the participants was measured while they were wearing light clothes without footwear, using a portable scale (to the nearest 0.1 kg). Waist circumference was measured at the midpoint between the lower margin of the last palpable rib and the top of the iliac crest, in an upright position [22]. Hip circumference was measured horizontally at the maximum circumference over the buttocks, (measured to the nearest 0.1 cm) [22]. Blood pressure (BP) (mmHg) was measured a total of three times with a three-minute pause, using an OMRON M6 blood pressure monitor. Mean systolic and diastolic BP were calculated from the average of the second and third measurements [22]. To investigate the fasting plasma glucose (FPG) and lipid profile, venous blood samples were collected into a lipid tube and glucose tube containing fluoride, and transported in cold boxes (between 2–8 °C) to the National Health Laboratory, Yangon—a reference laboratory of the Myanmar Ministry of Health—by boat and/or by car or motorcycle. FPG (mmol/L) concentration was determined by an enzymatic reference method with hexokinase, using reagents of COBAS from Roche Diagnostics, Indianapolis, IN. The serum concentration of TC and TG (mmol/L) was measured using an enzymatic colorimetric test with reagents of COBAS from Roche Diagnostics, Indianapolis, IN, all within three hours of collection.

#### 2.1.2. Oil 2016-Study

In a household-based cross-sectional study conducted in November and December 2016, vegetable oil samples were collected in urban and rural areas of the Yangon region. We used a multi-stage cluster sampling method in accordance with the WHO STEPs manual [22]. Eight townships (four urban, four rural) were randomly selected from the 45 townships of the Yangon region. Lists of wards (urban township units) and villages (rural township units) were collected from each township. Three wards and three villages were randomly selected from each of the urban and rural townships, respectively, equaling a total of 12 wards and 12 villages. In each ward and village, the sampling procedure was carried out based on the map of the village or ward. Using a table of random numbers, five streets or blocks were chosen randomly from each ward or village. From each selected block or street, five households were then randomly selected. A total of 114 out of 120 households (95%) participated. Samples (50 mL) of each type of oil used in food preparation in the households were collected, totaling 128 samples of vegetable oil. All oils were stored in sterile plastic containers, sealed airtight, and sent to Vitas analytical services, Oslo, Norway, for analysis of their fatty acid content.

Accurately weighed oil samples were added internal standard tritridecanoin (NuCheck Prep, INC., Elysian, MN, USA) and then dissolved in Hexane:Dichloromethane:Methanol (4:2:1) and well mixed. The dissolved samples were methylated with 3N Methanolic HCl at a temperature of 50 °C at 450 rpm for 16 h. The samples were cooled to room temperature and fatty acid methyl esters (FAMEs) were extracted to hexane followed by neutralization of the sample with 3N KOH. The samples were well mixed and centrifuged before being injected into a gas chromatography-flame ionization detection (GC-FID) system. GC analysis was performed on an Agilent 7890A system with a split/split less injector, 7683B Series automatic liquid sampler and FID (Agilent Technologies, Palo Alta, CA, USA). FAMEs were separated with a SP-2381 column (30 m × 0.25 mm × 0.25 µm film thickness), Supelco, Inc., Bellefonte, PA, USA. The fatty acid profile was assessed for each oil sample individually. In tables and analyses, we pooled the oil data according to type of oil.

### 2.2. Variables

Age was defined as the completed years of age. Educational level was defined by the highest educational level reached including the total number of school years, and classified into: no formal education (0 years), primary education (1–5 years), secondary education (6–11 years), and higher education (≥12 years). Daily income was calculated from the total income of the household divided by the number of household members ≥18 years old. Income was converted into United States Dollars (USD) from Myanmar Kyats. According to a World Bank report, the threshold values for poverty were defined as 1.90 USD/day (extreme poverty) and 3.10 USD/day (moderate poverty) [24]. High intake of fruit and vegetables was defined as five servings/day, according to WHO guidelines [22]. A standard serving means approximately 80 g of fruit or vegetable. Show cards were used for the approximation of serving size. Body mass index (BMI) was calculated as weight (kg) divided by height (m^2^).

#### Oil 2016-Study

Branded vegetable oil was defined as vegetable oil that was produced locally or abroad and sold in a sealed bottle with a brand name and manufacturing and expiry dates. The labeling of fatty acid composition is optional in Myanmar. Non-branded vegetable oil was defined as vegetable oil produced locally or abroad, not sold in a sealed bottle, and without a brand name, a manufacturing and expiry date, or a description of FA composition. Both branded and non-branded peanut oil and palm oil were sold. A mix of peanut and palm oil was sold non-branded. Sunflower oil, sesame oil, soybean oil, and olive oil were sold branded. Soybean, sunflower, and olive oils were imported. The intake of oil per participant (L/person/month) was defined as the monthly household oil consumption divided by the number of household participants. For branded vegetable oil, the volume of oil was counted from the bottle. For non-branded oils, the oil was bought in small amounts, e.g., 25 tical/day or 1 viss/time according to Myanmar measurement units. We converted tical or viss to liters (1 viss = 0.75 L; 1 viss = 100 tical).

### 2.3. Data Management and Statistical Analysis

The Epidata software (version 3.1, Epidata Association, Odense, Denmark) was used for double data entry and STATA/IC (version 15.1, StataCorp LP, College Station, TX, USA) was used for data analysis. In the Oil 2016-study, the FA contents of the various cooking oils used were presented as grams of FA per 100 g of oil (median), and total content of SFA, MUFA, and PUFA. For both studies, the proportion of participants using each type of oil in cooking was reported. The STEPS 2014-study was used to assess the association between the type of oil used (palm oil vs. peanut oil) and risk factors for NCDs. Complex survey data was declared using ‘svyset’, and the prefix command ‘svy’ was used in the analysis for both studies. The 2014 Myanmar census [25] was used for standardization by applying the different stages of sampling units of the study population. In separate linear regression analyses, the associations between the type of oil (palm oil vs. peanut oil) and BMI, WHR, BP, FPG, TC, and TG were estimated. Each of the analyses was based on a Directed Acyclic Graph (DAG) [26], in which age, location, education, income, and fruit and vegetable consumption were confounders, and thus adjusted for. Interaction was found between sex and the type of oil used, and analyses were thus stratified by gender. The associations indicate the total effect of cooking using palm oil vs. peanut oil on the respective risk factors.

### 2.4. Ethical Considerations

This study was conducted according to the guidelines of the Declaration of Helsinki. The proposals for the STEPS 2014-study were approved by the Ethical Committee, the Department of Health, the Ministry of Health, Myanmar (10/2013/749), and Norwegian Regional Committees for Medical and Health Research Ethics (2013/1088), and the proposals for the Oil 2016-study were approved by the Ethical Review Committee, the Department of Medical research, the Ministry of Health and Sports, The Republic of the Union of Myanmar (Ethics/DMR/2016/142), and Norwegian Regional Committees for Medical and Health Research Ethics (2016/379). All respondents gave their informed consent prior to their participation in the study.

## 3. Results

The proportion of women was higher in the Oil 2016-study than in the STEPS 2014-study. The proportions of participants with no formal education and who were living on less than 1.90 USD/day were higher in the Oil 2016-study than in the STEPS 2014-study. The urban–rural distribution was roughly the same in both studies.

**Figure 1:**
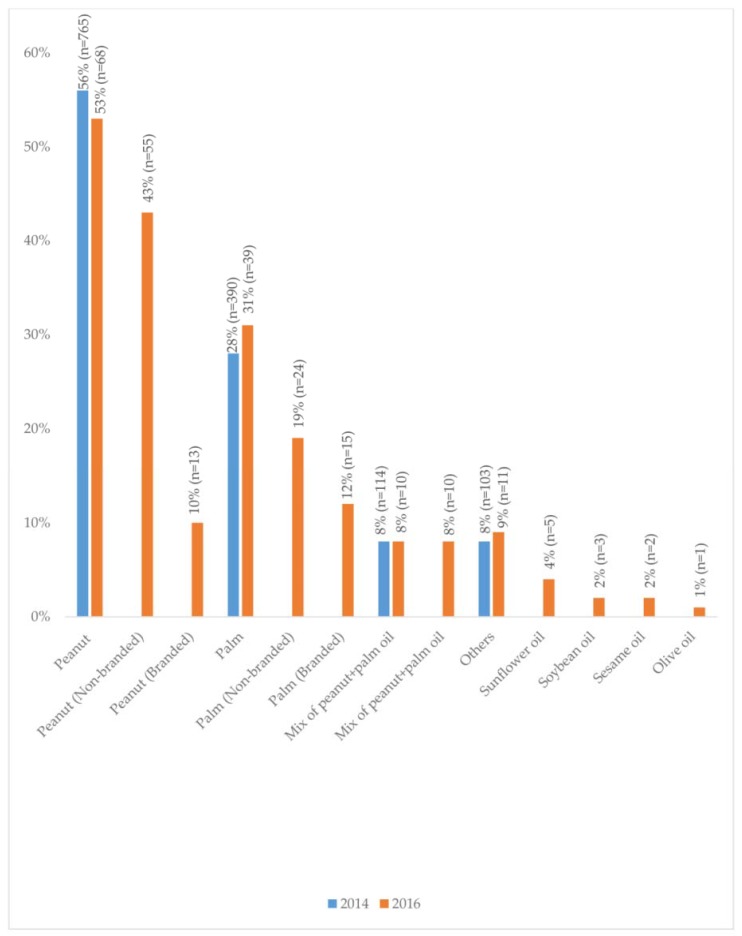
The proportion of participants using various vegetable oils for cooking purposes in the Yangon region between 2014 and 2016.

The oils most commonly used in cooking were peanut oil and palm oil, in both the STEPS 2014-study and the Oil 2016-study. In the Oil 2016-study, non-branded oils were more commonly used than branded ones, both for peanut oil (43% vs. 10%) and palm oil (19% vs. 12%) (Figure 1). Sunflower oil, soybean oil, sesame oil, and olive oil were used by a smaller proportion of the participants. The mean intake of vegetable oil was 1.31 L/person/month, with no differences between gender, location, education, or income. In the STEPS 2014-study, rural inhabitants, participants with no formal education and primary school completed, and participants with incomes of <1.90 USD/day, were more likely than others to use palm oil compared to peanut oil (Table 1).

Non-branded palm oil had the highest SFA content (36 g/100 g), followed by a mixture of peanut and palm oil (33 g/100 g), branded palm oil (32 g/100 g), and non-branded peanut oil (31 g/100 g) (Table 2). The high content of SFA in these oils was reflected in a high content of palmitic acid (C_16:0_), the major SFA in all types of oils. Stearic acid (C_18:0_) was the second major SFA in all oils, although it was present in substantially smaller amounts (3 g/100 g). Other SFAs were observed in trace amounts only. The content of gondoic acid (C_20:1, n-9)_ ranged from <1 g/100 g in non-branded palm oil to 0.7 g/100 g in soybean oil. Myristic acid (C_14:0_) was found in all oil types in the study. Regarding palm oil, the contents of SFA and PUFA were higher in non-branded oils than in branded oils (*p* value < 0.05).

The main MUFA in the oils was oleic acid (C_18:1, n-9_), the content of which was highest in olive oil (35 g/100 g) (Table 2). Linoleic acid (C_18:2, n-6_) was the main PUFA, the content of which was highest in sesame oil and lowest in non-branded palm oil. The content of linoleic acid was higher in branded oils than in non-branded oils. All types of oil contained trace amounts of trans fat (<0.1 g/100 g, not shown), with non-branded palm oil having the highest content (0.04 g/100 g).

Men using palm oil for cooking, compared to peanut oil, had −0.16 (95% CI = −0.30, −0.01) mmol/L lower mean fasting plasma glucose levels, and lower mean BMI (−1.35 (−2.22, −0.49) kg/m^2^). Women using palm oil, compared to peanut oil, had 3.55 (0.84, 5.65) mmHg higher mean diastolic blood pressure, 0.28 (0.07, 0.49) mmol/L higher mean levels of total cholesterol, and on average 0.16 (0.02, 0.30) mmol/L higher triglyceride levels (Table 3).

## 4. Discussion

Non-branded peanut oil was the most commonly used vegetable oil in the Yangon region, followed by non-branded and branded palm oils. The content of SFA was higher, and the content of PUFA lower, in non-branded palm oil than in branded palm oil. Using palm oil, compared to peanut oil, was associated with lower mean FPG levels and lower BMI in men; however, it was associated with higher levels of diastolic BP, TG, and TC in women. 

The strengths of this study include the use of the internationally accepted World Health Organization (WHO) STEPs methodology for both studies [22], the high response rates (85% in the STEPS 2014-study and 95% in the Oil 2016-study), and the random sampling of data from both urban and rural areas. Buddhist monks, nuns, military personnel, and institutionalized people were not included in this study, since their dietary patterns, physical activity levels, and health behaviors might be different from those of the general population, and thus might affect estimates. The participants were sampled to reflect the general population of Yangon. In a 2014 study in Yangon, the age-standardized prevalences of hypertension and hypercholesterolemia were previously reported to be 34.6% and 46.8%, respectively, while the prevalences of diabetes were reported to be 12.1% (urban population) and 7.1% (rural population) [17,18,19]. The consumption pattern of oils in the Yangon region was comparable in both survey years. However, the proportion of participants with no formal education and incomes <1.90 USD/day was higher in the Oil 2016-study. The proportion of women was higher in the Oil 2016-study than in the STEPS 2014-study. The main contribution of the Oil 2016-study is the fatty acid compositions of the oils used, which were not affected by the proportion of women in the study.

One limitation of the study was that we were not able to analyze the content of oils, or the associations between the use of different oils and NCD risk factors in the same study. Arachidonic acid (C_20:4_), eicosapentaenoic acid (C_20:5_), and docohexaenoic acid (C_22:6_) were not detectable in our oil samples. Thus, we do not have a full fatty acid profile of each oil. However, non-detectable amounts of these fatty acids are in accordance with studies reporting that eicosapentaenoic acid and docohexaenoic acid are mainly found in fish and seafood [27], whereas arachidonic acid is found in algae and plants, and not in vegetable oils [28]. There may have been some reduction in fasting plasma glucose and lipid levels following blood extraction; however, we transported the blood samples in a cold box to the National Health Laboratory within three hours for investigation, and the blood samples were investigated immediately in the laboratory on the same day. We did not analyze minor components of the vegetable oils, such as Vitamin E in peanut oil and carotenes in palm oil, which could have influenced our estimates. Vitamin E has a favorable effect on the reduction of FPG and systolic and diastolic BP [29,30], while carotenes reduce the risk of type II diabetes mellitus [30]. Moreover, we did not have information about whether the oils were refined or cold-pressed, which could affect the content of minor components in the oils. Adults and children were equally involved in the calculation of the amount of oil used per month. Thus, some variation in intake estimates may have occurred. The results cannot be generalized to the whole of Myanmar, since the consumption patterns of various cooking oils differ between regions and ethnic groups [20].

In agreement with the present study, peanut oil has previously been reported to be the preferred oil for cooking in Lower Myanmar, followed by palm oil [21]. The choice of oil for cooking depends on education level, income, consumer preference, taste, and health knowledge [21,31]. Palm oil is the cheapest oil, and is often chosen by low-income people [21]. This is in line with our findings that palm oil was most commonly used by participants with low educational level and income, among rural dwellers and among women. More than half of the population of Yangon (54%) has an income of <1.90 USD/per day [19], which may explain the widespread use of palm oil. A study of the consumer preference for vegetable oil in Myanmar in 2011 showed that peanut oil was rated highly regarding taste, aroma, nutrition status, and health benefits; however, it had low affordability [32]. Conversely, palm oil was regarded as affordable, but had low scores for taste, aroma, nutrition, and health benefits [32]. Oils rich in SFAs have high stability and are therefore suitable for cooking and short-term frying [1], which is a popular way to prepare food in Myanmar. In addition to price, this may be one of the reasons that palm and peanut oils are commonly used in this population.

Non-branded oils are cheaper than branded oils, and are most often the choice of low-income people. However, very few studies have been carried out on the content of these oils, and how they differ from their branded counterparts, along with potential health consequences. Non-branded palm oil is sold from large unlabeled tanks, sometimes mixed with peanut oil. Peanut oil is a local product, and was purchased as either branded or non-branded. It is possible that non-branded peanut oil sold as such is also mixed with palm oil, which is cheaper. The slightly less favorable FA composition of non-branded peanut oil compared to branded peanut oil observed in our study could indicate some degree of such a mix. Our results confirmed that non-branded oils have a less favorable fatty acid content than branded oils, with higher contents of SFAs and trans fat, and lower contents of PUFA. This could add to the disproportionate burden of health problems experienced by people with low socioeconomic status [33], although more research on the difference between branded and non-branded oils is still needed.

The fatty acid profile of peanut oil (both branded and non-branded) measured in our study deviates from that reported by United States Department of Agriculture (USDA) [34]. Possible explanations for this include the poor-quality production machines in the majority of oil mills in the Yangon region [35]. Moreover, non-branded oil is sold in different plastic bottles at local markets [35]. These conditions deteriorate the quality of vegetable oils. The adulteration of crude oil with low quality or reused oil [36] is also a possible contributor to the deviation from the standard quality of peanut oil. Furthermore, climatic conditions such as lower temperature, mature status of peanut seeds, genotype, land location, and interaction between these factors are major influencing factors of the fatty acid content of peanut oils [37,38]. This is another possible explanation for our results. Our findings support the observation that the SFA content of non-branded peanut oils is higher than that of branded peanut oils, and also found a similar pattern in palm oils.

Palm oil has long been considered as harmful to health, mainly due to its high SFA content [6]. However, results from recent studies have modified this notion, partly by showing that different SFAs may have different effects on cardiovascular health risk [39]. Studies exploring the harm or benefit of using palm oil in cooking have obtained various results [40]. The results appear to depend on what kind of fat palm oil is compared to and the total amount of fat in the diet, among various other factors. A meta-analysis of clinical trials reported that palm oil consumption triggered an increase in LDL levels compared to the consumption of vegetable oils with low SFA, but increased HDL levels compared to the consumption of vegetable oil with a high content of trans fat [6].

The results of our study suggest that using palm oil, compared to peanut oil, was associated with some lower NCD risk factor levels in men, and with some higher NCD risk factor levels in women. Men who consumed palm oil had lower levels of BMI and FPG compared to peanut-oil consumers. Women who consumed palm oil had higher mean levels of diastolic BP, TC, and TG, compared to those who consumed peanut oil. The association between the use of palm oil and higher levels of TC among women is in line with research showing that a high concentration of palmitic and myristic acid, together with lauric acid (C_12:0_), is associated with higher blood cholesterol level [7]. The associations between NCD risk factor levels and the use of palm oil varied between women and men, and also after adjustment for possible confounders. We adjusted the analyses for the intake of fruit and vegetable; however, it is possible that the intake of different kinds of food accompany the oil consumed by women and men, which differently affects the risk of NCDs. In Myanmar, men are commonly breadwinners who work outside the house [19], and may be less exposed to foods cooked at home. The association between the intake of oils and NCD risk is influenced not only by the type of oil, but also by the amount consumed [41]. However, in our study, the amount of oil consumed did not vary with socioeconomic position, location, or gender. Other unmeasured confounders could explain the observed sex differences in the association between the use of oil and NCD risk factors. New studies are warranted to explore this further. It is challenging to give general advice on whether or not to prefer peanut oil over palm oil based on our results, since the findings differ between genders. Peanut oil appears to be a better choice for women, with the results suggesting a lower diastolic blood pressure for female peanut oil consumers, in line with previous literature [42].

Oils rich in MUFA and PUFA were used by very few participants in our study. Olive oil was the oil that was most rich in oleic acid in our study, as also reported in a French study [43]. A high concentration of MUFA, especially oleic acid, could lead to a reduction in TC and LDL levels, and have a preventative effect for cardiovascular attacks [44]. Linoleic acid was the main PUFA in all oils, whereas other PUFA were found in trace amounts only. Sesame oil had the highest content of PUFA, especially linoleic acid (C_18:2, n-6_). A previous Myanmar nationwide study reported that using sesame oil, compared to peanut oil, was associated with a lower risk of hypertension [45]. Sesame oil has previously been reported to have a supportive role in reducing blood pressure in hypertensive patients [9], while a high consumption of PUFA has been associated with low FPG levels [11]. An increased consumption of vegetable oils rich in PUFA could have positive health effects in Myanmar, and should thus be advocated.

## 5. Conclusions

This article reports the FA content of commonly consumed vegetable oils in the Yangon region, Myanmar. Non-branded oils were most commonly used in food preparation, and non-branded palm oil had a less favorable fatty acid composition compared to branded palm oil. The lower price of non-branded oils and limited information about how to make healthy choices could be barriers to changing to healthier alternatives. The adulteration of original oils with low-quality oils, and low-quality production techniques, could contribute to substandard quality in vegetable oils. Appropriate interventions, such as the enforced labeling of content on oil bottles, encouraging the regulation of non-branded oils, and providing information on how to choose healthy oils, could help to reduce the intake of oils rich in SFA and trans fats, and help to reduce the risk of NCD in Myanmar. Using palm oil, compared to peanut oil, was associated with a more favorable NCD risk profile among men, but a less favorable risk profile among women. Further research into the level of vegetable oil consumption and food preparation, with a focus on sex differences, is warranted.

## Figures and Tables

**Table 1:** Sociodemographic characteristics of urban and rural 25–74-year-old participants from the Yangon region, Myanmar, between 2014 and 2016.

	2014 STEP Study				Oil 2016 Study			
	Total (*n* = 1372) *N* (%)	Peanut Oil User (*n* = 765) *N* (%)	Palm Oil User (*n* = 390) *N* (%)	*p* Value *	Total (*n* = 114) *N* (%)	Peanut Oil User (*n* = 61) *N* (%)	Palm Oil User (*n* = 35) *N* (%)	*p* Value *
Gender				0.09				0.47
Male	681 (49.6)	376 (49.2)	212 (54.4)		30 (26.3)	20 (32.8)	9 (25.7)	
Female	691 (50.4)	389 (50.9)	178 (45.6)		84 (73.7)	41 (67.2)	26 (74.3)	
Age group				0.01				0.80
25–34	259 (18.9)	144 (18.8)	79 (20.3)		19 (16.7)	7 (11.5)	6 (17.1)	
35–44	316 (23.0)	162 (21.2)	99 (25.4)		23 (20.2)	12 (19.7)	9 (25.7)	
45–54	327 (23.8)	168 (22.0)	101 (25.9)		35 (30.7)	18 (29.5)	10 (28.6)	
55–64	306 (22.3)	181 (23.7)	80 (20.5)		28 (24.6)	19 (31.2)	8 (22.9)	
65–74	164 (12.0)	110 (14.4)	31 (8.0)		9 (7.9)	5 (8.2)	2 (5.7)	
Education level				<0.001				0.07
No formal education	86 (6.3)	28 (3.7)	47 (12.1)		26 (22.8)	12 (19.7)	12 (34.3)	
Primary education	641 (46.8)	300 (39.2)	257 (65.9)		37 (32.5)	17 (27.9)	14 (40.0)	
Secondary education	423 (30.8)	270 (35.3)	78 (20.0)		27 (23.7)	17 (27.9)	6 (17.1)	
Higher education	222 (16.2)	167 (21.8)	8 (2.1)		24 (21.1)	15 (24.6)	3 (8.6)	
Location				<0.001				0.004
Urban	693 (50.5)	464 (60.7)	110 (28.2)		55 (48.2)	36 (59.0)	10 (28.6)	
Rural	679 (49.5)	301 (39.4)	280 (71.8)		59 (51.8)	25 (41.0)	25 (71.4)	
Daily income				<0.001				0.23
<1.9 USD/day	743 (54.2)	359 (46.9)	266 (68.2)		71 (62.3)	40 (65.6)	19 (24.3)	
1.9–3.09 USD/day	252 (18.4)	144 (18.8)	67 (17.2)		22 (19.3)	10 (16.4)	11 (31.4)	
≥3.1 USD/day	298 (21.7)	205 (26.8)	41 (10.5)		21 (18.4)	11 (18.0)	5 (14.3)	

* Chi-square test. USD: United States Dollars.

**Table 2:** Median values and interquartile range (IQR) of the fatty acid composition of vegetable oils (in grams of fatty acids/100 g of oil) from the Yangon region, 2016.

Fatty Acid Classification	Peanut Oil (Non-Branded)	Peanut Oil (Branded)	Palm Oil (Non-Branded)	Palm Oil (Branded)	Mix of Peanut + Palm Oil	Sunflower Oil	Soybean Oil	Sesame Oil	Olive Oil
	Median (IQR)								
C_12:0_	0.2 (0.3)	0.2 (0.2)	0.2 (0.1)	0.2 (0.2)	0.2 (0.1)	<0.05 (0.1)	<0.05 (0.1)	<0.05 (<0.05)	<0.05 (0)
*p* value *	0.302		0.171						
C_14:0_	0.7 (0.9)	0.6 (0.8)	0.9 (0.1)	0.7 (0.8)	0.8 (0.2)	0.1 (0.3)	0.1 (0.6)	<0.05 (<0.05)	<0.05 (0)
*p* value *	0.259		0.006						
C_16:0_	26.5 (20.1)	24.7 (19.8)	31.2 (21.4)	27.1 (21.4)	28.1 (6.4)	9.2 (12.5)	11.0 (15.5)	9.2 (0.6)	9.5 (0)
*p* value *	0.496		0.018						
C_16:1_	0.1 (<0.05)	0.1 (0.1)	0.2 (0.1)	0.1 (0.1)	0.2 (<0.05)	0.1 (0.1)	0.1 (0.1)	0.1 (<0.05)	0.1 (0)
*p* value *	0.365		0.190						
C_17:0_	0.7 (<0.05)	0.1 (<0.05)	0.1 (<0.05)	0.1 (<0.05)	0.1 (0.1)	0.1 (<0.05)	0.1 (<0.05)	<0.05 (<0.05)	0.1 (0)
*p* value *	0.418		0.484						
C_18:0_	2.9 (0.4)	2.9 (0.2)	2.9 (0.3)	2.9 (0.5)	3.0 (0.3)	2.8 (0.3)	3.2 (0.5)	3.9 (0.1)	2.9 (0)
*p* value *	0.952		0.343						
C_18:1, n-9_	32.4 (4.1)	31.1(2.7)	32.2 (4.2)	32.8 (4.9)	32.6 (1.0)	34.7 (7.7)	28.1 (20.3)	31.9 (2.0)	35.1 (0)
*p* value *	0.377		0.517						
C_18:1, c-11_	0.6 (0.1)	0.6 (0.3)	0.6 (0.1)	0.6 (0.2)	0.6 (0.1)	0.6 (0.1)	0.5 (0.6)	0.7 (0.1)	0.4 (0)
*p* value *	0.653		0.658						
C_18:2, n-6_	13.3 (13.3)	17.1 (17.2)	9.2 (0.1)	15.1(13.7)	13.6 (6.6)	22.0 (29.6)	26.6 (28.5)	32.9 (7.3)	22.4 (0)
*p* value *	0.358		0.017						
C_18:3, n-3_	0.2 (0.3)	0.2 (0.3)	0.2 (0.1)	0.2 (0.1)	0.3 (0.5)	0.2 (0.1)	0.1 (0.2)	0.2 (0.1)	0.0 (0)
*p* value *	0.494		0.865						
C_20:0_	0.3 (0.3)	0.3 (0.3)	0.3 (0.1)	0.3 (0.3)	0.3 (<0.05)	0.5 (0.5)	0.5 (1.1)	0.6 (0.2)	1.1 (0)
*p* value *	0.942		0.053						
C_20:1, n-9_	0.1(0.4)	0.2 (0.6)	0.1 (0.5)	0.2 (0.5)	0.1 (<0.05)	0.3 (<0.05)	0.7 (4.9)	0.2 (0.2)	0.7 (0)
*p* value *	0.229		0.057						
C_20:2, n-6_	<0.05 (<0.05)	<0.05 (<0.05)	<0.05 (<0.05)	<0.05 (<0.05)	<0.05 (<0.05)	<0.05 (<0.05)	<0.05 (<0.05)	0.1 (<0.05)	0.1 (<0.05)
*p* value	0.986		0.879						
C_22:0_	0.2 (0.6)	0.3 (2.0)	0.1 (<0.05)	0.3 (0.7)	0.1 (0.1)	0.6 (0.1)	0.6 (2.2)	0.1 (0.2)	1.9 (0)
*p* value *	0.357		0.026						
C_24:0_	0.1 (0.2)	0.1 (0.8)	0.1 (<0.05)	0.1 (0.2)	0.1 (0.1)	0.2 (<0.05)	0.3 (0.9)	0.3 (0.4)	0.7 (0)
*p* value *	0.463		0.031						
Total SFA	31.5 (17.2)	29.4 (17.6)	36.1 (4.8)	31.5 (18.2)	32.7 (6.2)	15.9 (14.2)	19.4 (16.7)	14.2 (1.1)	16.3 (0)
*p* value *	0.467		0.018						
Total MUFA	33.6 (4.2)	34.2 (2.7)	33.0 (4.2)	33.9 (5.0)	33.7 (1.1)	35.7 (8.0)	32.9 (15.2)	33.0 (2.0)	36.2 (0)
*p* value *	0.425		0.410						
Total PUFA	13.5 (13.2)	17.8 (17.2)	9.3 (0.9)	15.3 (13.6)	14.1 (7.0)	22.1 (29.9)	26.7 (28.7)	33.1 (7.4)	22.5 (0)
*p* value *	0.343		0.023						
Total FA	79.6 (5.9)	78.9 (3.3)	78.6 (7.8)	80.0 (9.5)	80.8 (2.5)	80.4 (7.6)	77.5 (7.6)	80.3 (4.2)	75.0 (0)
*p* value *	0.851		0.946						

* *t* test, difference between branded and non-branded oil. SFA—saturated fatty acid, MUFA—monounsaturated fatty acid, PUFA—polyunsaturated fatty acid, FA—fatty acid.

**Table 3:** The association between using palm oil in cooking compared to peanut oil, and various risk factors for non-communicable diseases, among 25–74-year-old citizens in the Yangon region between 2013–2014, and linear regression analysis.

	Crude	Adjusted for Age	Adjusted for Age, Location	Adjusted for Age, Location, Education, Income	Adjusted for Age, Location, Education, Income, Fruit and Vegetable Intake
	β (95% CI)	β (95% CI)	β (95% CI)	β (95% CI)	β (95% CI)
**Male**					
Body mass index (kg/m^2^)	−2.11 (2.96, −1.26) ***	−2.05 (−2.84, −1.26) ***	−1.79 (−2.67, −0.92) **	−1.34 (−2.18, −0.50) **	−1.35 (−2.22, −0.49) **
Waist-hip ratio	−0.11 (−0.24, 0.02)	−0.09 (−0.22, 0.04)	−0.10 (−0.22, 0.03)	−0.05 (−0.17, 0.07)	−0.05 (−0.17, 0.07)
Fasting plasma glucose (mmol/L)	−0.39 (−0.54, −0.25) ***	−0.26 (−0.42, −0.11) **	−0.11 (−0.28, 0.07)	−0.16 (−0.32, −0.00) *	−0.16 (−0.30, −0.01) *
Systolic blood pressure (mmHg)	−1.44 (−5.48, 2.60)	0.61 (−3.84, 5.06)	0.83 (−3.24, 4.90)	1.15 (−3.67, 5.98)	1.21 (−3.46, 5.88)
Diastolic blood pressure (mmHg)	−1.34 (−3.83, 1.17)	−0.96 (−3.49, 1.57)	−0.55 (−2.75, 1.66)	−0.38 (−3.06, 2.31)	−0.30 (−2.80, 2.20)
Total cholesterol (mmol/L)	−0.37 (−0.55, −0.19) **	−0.33 (−0.53, −0.13) **	−0.21 (−0.42, −0.01) *	−0.19 (−0.40, 0.03)	−1.89 (−0.41, 0.03)
Triglycerides (mmol/L)	−0.23 (−0.52, 0.06)	−0.22 (−0.52, 0.08)	−0.14 (−0.43, 0.16)	−0.13 (−0.45, 0.19)	−0.13 (−0.45, 0.19)
**Female**					
Body mass index (kg/m^2^)	−0.32 (−0.47, −1.62)	0.17 (−1.77, 2.10)	0.40 (−1.44, 2.24)	0.68 (−1.25, 2.61)	0.63 (−1.13, 2.39)
Waist–hip ratio	0.06 (−0.01, 0.13)	0.06 (0.01, 0.11)	0.04 (−0.02, 0.10)	0.05 (−0.02, 0.11)	0.05 (−0.03, 0.12)
Fasting plasma glucose (mmol/L)	−0.37 (−0.85, 0.11)	−0.36 (−0.80, 0.09)	−0.27 (−0.81, 0.09)	−0.45 (−0.99, 0.09)	−0.50 (−1.10, 0.08)
Systolic blood pressure (mmHg)	3.00 (−1.37, 7.37)	3.22 (−0.22, 6.65)	3.93 (0.22, 7.65) *	2.54 (−0.77, 5.84)	2.64 (−0.82, 6.11)
Diastolic blood pressure (mmHg)	3.28 (0.50, 6.07) *	3.33 (0.88, 5.78) *	3.86 (1.21, 6.51) **	3.08 (0.73, 5.44) *	3.25 (0.84, 5.65) *
Total cholesterol (mmol/L)	0.29 (0.03, 0.55) *	0.30 (0.09, 0.51) **	0.33 (0.14, 0.52) **	0.31 (0.09, 0.52) **	0.28 (0.07, 0.49) *
Triglycerides (mmol/L)	0.12 (−0.10, 0.34)	0.16 (−0.07, 0.32)	0.18 (0.02, 0.34) *	0.17 (0.03, 0.31) *	0.16 (0.02, 0.30) *

* *p* value <0.05, ** *p* value <0.01, *** *p* value <0.001.
